# A qualitative study of the barriers and facilitators impacting the implementation of a quality improvement program for emergency departments: SurgeCon

**DOI:** 10.1186/s12913-024-11345-w

**Published:** 2024-07-27

**Authors:** Nahid Rahimipour Anaraki, Meghraj Mukhopadhyay, Jennifer Jewer, Christopher Patey, Paul Norman, Oliver Hurley, Holly Etchegary, Shabnam Asghari

**Affiliations:** 1 Centre for Rural Health Studies, Faculty of Medicine, Memorial University of Newfoundland, St. John’s, NL A1B 3V6 Canada; 2 Faculty of Business Administration, Memorial University of Newfoundland, St. John’s, NL A1B 3V6 Canada; 3 Discipline of Family Medicine, Faculty of Medicine, Memorial University of Newfoundland, St. John’s, NL A1B 3V6 Canada; 4 Eastern Health, Carbonear Institute for Rural Reach and Innovation By the Sea, Carbonear General Hospital, Carbonear, NL A1Y 1A4 Canada; 5 Faculty of Medicine, Memorial University of Newfoundland, St. John’s, NL A1B 3V6 Canada; 6 Discipline of Family Medicine, Faculty of Medicine, Faculty of Medicine Building, Memorial University of Newfoundland, 300 Prince Philip Drive, St. John’s, Newfoundland, A1B 3V6 Canada

**Keywords:** SurgeCon, Quality improvement program, Barriers, Facilitators, Pre-implementation, Emergency department

## Abstract

**Background:**

The implementation of intervention programs in Emergency Departments (EDs) is often fraught with complications due to the inherent complexity of the environment. Hence, the exploration and identification of barriers and facilitators prior to an implementation is imperative to formulate context-specific strategies to ensure the tenability of the intervention.

**Objectives:**

In assessing the context of four EDs prior to the implementation of SurgeCon, a quality improvement program for ED efficiency and patient satisfaction, this study identifies and explores the barriers and facilitators to successful implementation from the perspective of the healthcare providers, patients, researchers, and decision-makers involved in the implementation.

**Settings:**

Two rural and two urban Canadian EDs with 24/7 on-site physician support.

**Methods:**

Data were collected prior to the implementation of SurgeCon, by means of qualitative and quantitative methods consisting of semi-structured interviews with 31 clinicians (e.g., physicians, nurses, and managers), telephone surveys with 341 patients, and structured observations from four EDs. The interpretive description approach was utilized to analyze the data gathered from interviews, open-ended questions of the survey, and structured observations.

**Results:**

A set of five facilitator-barrier pairs were extracted. These key facilitator-barrier pairs were: (1) management and leadership, (2) available resources, (3) communications and networks across the organization, (4) previous intervention experiences, and (5) need for change.

**Conclusion:**

Improving our understanding of the barriers and facilitators that may impact the implementation of a healthcare quality improvement intervention is of paramount importance. This study underscores the significance of identifing the barriers and facilitators of implementating an ED quality improvement program and developing strategies to overcome the barriers and enhance the facilitators for a successful implementations. We propose a set of strategies for hospitals when implementing such interventions, these include: staff training, champion selection, communicating the value of the intervention, promoting active engagement of ED staff, assigning data recording responsibilities, and requiring capacity analysis.

**Trial registration:**

ClinicalTrials.gov. NCT04789902. 10/03/2021.

**Supplementary Information:**

The online version contains supplementary material available at 10.1186/s12913-024-11345-w.

## Introduction

### Research motivation

Wait times and overcrowding in emergency departments pose a severe national challenge for Canada as it has one of the highest wait times as compared to similarly industrialized countries [1]. This issue has persistently worsened as the number of emergency department (ED) visits in Canada has been increasing steadily over the past decade. From 2010–2011 to 2019–2020, the number of ED visits increased from approximately 6.7 million to 7.6 million, representing an average annual increase of 1.2%. Furthermore, the number of reported ED visits rose to almost 14.9 million in 2021–2022 from 11.7 million in 2020–2021[2]. The typical duration of a visit to an ED is around 3.5 h, which poses a risk to patients since prolonged waiting times in EDs have been associated with sub-optimal patient outcomes [3–9], and to the increased likelihood of adverse events [10]. To address this issue, SurgeCon, a quality improvement program, was devised to address the lack of integration, sustainability and logistical issues which negatively impact wait times in EDs [11–13]. SurgeCon delivers its quality improvement program through a department level management platform that encompasses three key elements: the installation and configuration of a tailored eHealth system, organizational restructuring, and the establishment of a patient-centric environment. SurgeCon aims to go beyond simply improving wait times; it seeks to optimize ED efficiency while providing a high standard of care for patients and promoting communication among clinicians.

Implementation of such a multidimensional quality improvement program in the dynamic and complex organizational structure of EDs, requires exploring barriers and facilitators prior to the respective implementation to formulate a set of strategies to enhance facilitators and overcome barriers, which may lead to a redesign of the program itself. Previous research in this area either lack the inclusion of strategies to overcome barriers or solely concentrate on eHealth adoption and implementation while neglecting considerations towards restructuring the organization of EDs and improving communication among clinicians. Barriers are factors that inhibit the implementation of practice change [14], while facilitators are factors that make the implementation easier [15]. Schreiweis et al. (2019) identified 76 barriers and 268 facilitators of implementation of eHealth services in health care out of 38 articles published between 2007 to 2018 from 12 different countries [16]. The most frequent barriers were categorized in three categories: individuals (e.g., poor digital health literacy), environmental and organizational (e.g., problems with financing eHealth solutions), and technical (e.g., lack of necessary devices). Also, some of the most stated facilitators were as follows: individuals (e.g., improvement in communication), environmental and organizational (e.g., involvement of all relevant stakeholders), and technical (e.g., ease of use). A limited number of studies have been conducted in an ED setting. For instance, Gyamfi et al., (2017) along with Kirk et al. (2016) and MacWilliams et al. (2017) explored relevant facilitators (e.g., capacity building, involvement and moral support of management and implementers, training and motivation, and environmental context and resources) and barriers (e.g., financial resources, data entry errors, shortage of human resources, and logistical constraints) that influence the implementation of eHealth services (e.g., Electronic Medical Records and screening tools) in EDs in Denmark, Ghana, and Canada (Ontario and Nova Scotia) [17–19]. Gyamfi et al. (2017) and Kirk et al. (2016) utilized semi-structured interviews, while MacWilliams et al. (2017) utilized focus groups for their data collection (these findings were then thematically analyzed) [17–19]. MacWilliams et al. (2017) also proposed suggestions to overcome barriers to implementation of Electronic Medical Records in EDs such as providing sufficient logistics (e.g., computers and accessories, reliable internet), rewarding staff, and regular staff training [19].

The aforementioned literature, along with other related literature, does not encompass the exploration of barriers and facilitators prior to the implementation of a large-scale quality improvement program that targets not only technical (i.e., eHealth system), but also structural (i.e., restructuring the ED organization and fostering patient-centric environment) and human (i.e., promoting communication across clinicians) aspects of the healthcare system. In fact, the objective of the quality improvement program in this study is not only to improve wait time, but also patient satisfaction, provider satisfaction, and the quality of care provided in EDs. As such, the SurgeCon program is connected to multiple dimensions related to patient outcomes within EDs. This study aims to explore barriers and facilitators prior to the implementation of SurgeCon in two rural and two urban Canadian EDs and formulate a set of strategies to overcome barriers and enhance facilitators. The findings identify areas of change for practitioners and policymakers [20]. This study is based on in-depth semi-structured interviews with clinicians, telephone interviews with patients, and structured observations in four EDs.

### SurgeCon: a quality improvement program

SurgeCon includes three components: implementing an eHealth system to automate an action-based surge capacity plan, restructuring the ED organization and workflow, and fostering a more patient-centric environment. SurgeCon’s eHealth system predicts surge levels which sets appropriate automated workflows in motion to enact proactive measures to improve patient flow and associated outcomes. Crucially, eHealth interventions are generally reported to have a positive impact on patient care [21] wherein its impact ranges from an increase in the availability of patient information, enhanced communication between healthcare workers, improved healthcare accessibility, and reduced patient wait times [13, 22, 23]. The evolution of eHealth services can be attributed to solving critical challenges faced by healthcare institutions around the world, such as wait times and overcrowding which are significant challenges for EDs globally [24, 25]. In addition to SurgeCon's eHealth system, a comprehensive approach to improving ED efficiency is also provided by including a patient flow course for frontline nurses and physicians. This course focuses on patient-centeredness and introduces process improvement strategies such as enhancing collaboration between physicians and mid-level providers like nurse practitioners, prioritizing stable patients based on factors beyond just acuity, and aiming to decrease the duration between a patient's arrival and their first assessment by a physician. Furthermore, SurgeCon's implementation process aims to improve the patient experience while in the department. This involves identifying problem areas that could negatively impact a patient's physical and mental well-being, such as their comfort level, ease of navigation, cleanliness of the department, clutter in the ED, and other related factors.

## Methods

### Study design

We employed a mixed-method approach at the technique level, incorporating semi-structured interviews, a structured questionnaire, and structured observation to collect data. To analyze the data, we adopted an interpretive description approach, as outlined by Thorne et al. (1997) [26]. This approach entails situating the findings within the current body of knowledge and drawing upon the contributions of other scholars, as highlighted by Mitchell and Cody (1993) [27]. This study aims to provide rich descriptive information on the key barriers and facilitators based on the language of the people involved, which inherently requires some degree of interpretation. The existing knowledge is not an organizing structure, rather, it serves as a foundational framework, providing a starting point and acts as an appropriate platform “upon which the design logic and the inductive reasoning in interpreting meanings within the data can be judged” [26].

### Study context

The implementation of SurgeCon in this study follows a stepped-wedge cluster trial design, specifically focusing on EDs within Category A hospitals. These hospitals offer round-the-clock physician coverage in their EDs. All the hospitals involved in the study are located within the same jurisdiction, operating under the same governance and management structure. The two rural intervention sites in this trial are similar in size, each with a capacity of 8 ED beds. They have a staff roster consisting of approximately 6–10 physicians and 12 nurses, divided into two teams that work on rotating schedules.

One of the urban sites is an acute care facility that provides services to the entire province. The other urban site has 15 beds and shares a physician roster of approximately 40 physicians with the other urban site. Each ED at the urban sites is staffed with 55 and 70 nurses, respectively. Both urban sites offer a wide range of inpatient and outpatient services, including several tertiary services (Table 1).

**Table 1:** Intervention Site Information (2021)

Emergency Departments	# of ED Physicians	# of ED Nurses	# of ED Visits/Month	# of ED Beds	LOS (Minutes—Monthly Avg)
Rural 1	6	12	1700	8	130
Rural 2	6	12	1800	8	150
Urban 1	40	70	4500	15	240
Urban 2	40	55	3200	23	210

### Data collection

Prior to the implementation of the SurgeCon intervention we conducted semi-structured, in-depth interviews with a total of 31 clinicians. This cohort comprised of 20 clinicians from rural EDs and 11 from urban EDs. This included 12 nurses, 9 physicians, 7 managers, 2 patient care facilitators, and 1 program coordinator with 1 to 32 years of work experience in EDs with 69% of participants identifying as female. The interview questions were informed by the Consolidated Framework for Implementation Research (CFIR), Organization Readiness for Knowledge Translation (OR4KT) domains, and the clinical/content expertise of the team. The recruitment continued until data saturation was achieved [28].

Data on patient satisfaction and patient-reported experiences with ED wait times were collected through telephone surveys that took place from March 1, 2021, to August 31, 2021. In total, 341 patients who visited one of the four selected EDs were interviewed, with 136 coming from rural EDs and 205 from urban EDs. The mean age was 55.7 (SD = 16.8) with 66% of participants identifying as female. We analyzed open-ended questions that specifically targeted patients' experiences while receiving care at the selected EDs and gathered their suggestions for improving the ED environment. Patients' insights confirmed our findings regarding resources, communication, and the necessity for change. The interview guide adapted questions from previously validated questionnaires which include the *Ontario Emergency Department Patient Experience of Care Survey*, the *CIHI Canadian Patient Experiences Survey*, the *Press Ganey Emergency Department Survey*, and the *NHS Accident and Emergency Department Questionnaire*.

Structured observations were conducted by research team members who were also healthcare staff and had special permission to visit each of the sites which were locked-down and only accessible to authorized ED personnel and patients due to COVID-19 pandemic restrictions. A ‘Site Assessment Checklist’ was used to assess each of the four EDs in terms of the ED’s available resources (e.g., medical, human, and technological), staff communication, pervious experiences of intervention, staff readiness and tension for change. The checklist was developed through a Delphi approach which included the input of research team members, ED staff, and patients who selected key criteria to assess the EDs.

The data collected and referenced in this analysis stems from an innovative pragmatic cluster randomized trial designed to evaluate the effects of SurgeCon, an ED management platform, on wait times and patient satisfaction. The subset of data that was considered relevant to our analysis was collected from March 2021 to December 2022. All data used in this study were collected prior to the implementation of SurgeCon at the four EDs selected for the cluster randomized trial. Even though each dataset was gathered and analyzed independently, they were considered complementary to each other instead of being mutually exclusive.

### Data analysis

Data from in-depth interviews, surveys, and structured observations was analyzed according to an interpretative description approach, while utilizing constant comparative analysis. Each set of data was repeatedly read by a qualitative researcher to comprehend the overall phenomena with questions such as “what is happening here? and “what am I learning about this?”, to become familiar with the data, to identify the potential themes or patterns and to achieve a broader insight about the phenomena [26, 28–30]. The data was then coded in a broad manner and continually compared and examined for similarities, differences, and relationships to help formulate major themes. A set of five facilitator-barrier pairs was extracted in this study.

All stages in the coding process were conducted by a qualitative researcher and were then categorically reviewed by members of the team to reach a consensus. The data analysis process started with the exploration of semi-structured interview data, which then progressed to include structured observation, and ended with the comprehensive analysis of the data gathered through surveys. Data extracted from semi-structured, in-depth interviews with clinicians served as the primary source for exploring barriers and facilitators before implementation. However, structured observations and survey data were integrated to offer additional clarity and act as auxiliary and confirmatory sources. Data collected from different stakeholders produced complementary results that captured multidimensional interpretations of the topic. The integrated blend of findings collected from various stakeholders through disparate methods not only explains multiple dimensions of the phenomena but also targets different audiences. In this study, data triangulation (gathering data at different times from various sources), investigator triangulation (multiple researchers study the topic of interest), and methodological triangulation (utilizing multiple methods) were utilized as cross-validation checks [31, 32].

### Findings

**Fig. 1:**
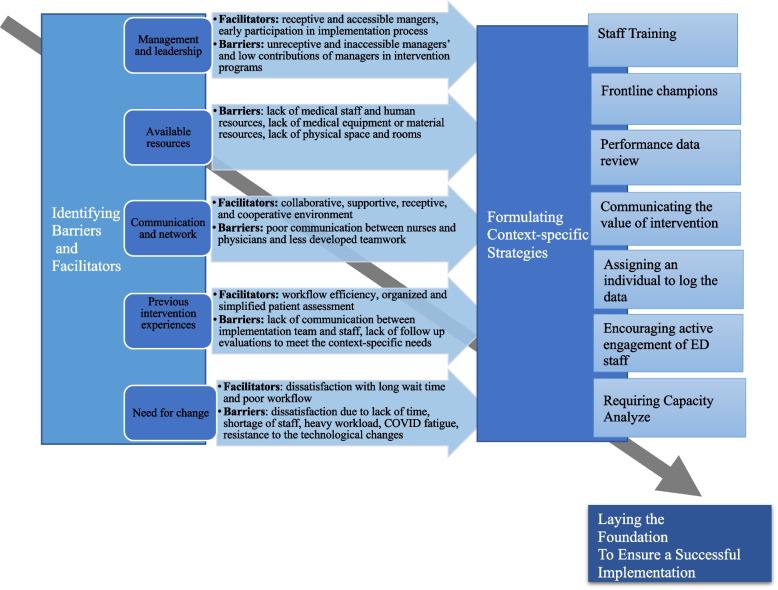
Process of Identifying Barriers and Facilitators toward Formulating Strategies

The barriers and facilitators to the implementation of SurgeCon fell into five themes, each of which plays a dual role of a barrier and facilitator (see Fig. 1). These key pairings were: (1) management and leadership, (2) available resources, (3) communications and networks across the organization, (4) previous intervention experiences, and (5) need for change. No significant differences were observed in terms of barriers and facilitators between the groups (i.e., rural and urban EDs) or among providers, patients, and observer inputs. While observer inputs provided insight on all categories, the patients’ input had the most influence on the following categories; available resources, communications and networks, and the need for change. In the following sections, we discuss each of these barrier and facilitator pairs.

### Management and leadership

The overarching management and leadership EDs was anticipated to be one of the most important facilitators of the SurgeCon implementation. Having a receptive, accessible, and supportive senior manager who is continually engaged with all aspects of the transition phase paired with an effective management system where the staff are involved in the decision-making process, was perceived to stimulate positive managerial-clinical communications along with an increasing likelihood for the positive reception of an implementation program. Active early involvement, support, and engagement of managers in two EDs were deemed crucial facilitators to fostering a nurturing and motivating environment that encourages physicians and nurses to proactively engage in the implementation process. Data from the observations served as confirmation of the involvement of both management and staff as well.

*“I can converse openly and there is an open-door policy. Furthermore, just in terms of communication, there is always a timely response and the manager is very proactive”* [Healthcare provider]

*“The site manager, the direct manager of the staff, comes every morning to the department to see what was happening last night. If there is any new issue, [the manager offers assistance and any logistical resolutions] that can be done or offered immediately. Additionally, they have free access to the director and to the manager through email. The manager’s office is just a few meters away from them, so they can just reach them at any time. For the doctors, the situation is also the same”* [Healthcare provider]

However, management and leadership could also pose barriers to a successful implementation. Barriers such as low manager participation and contribution, unreceptive and inaccessible managers, low staff autonomy and involvement in decision-making, and the lack of staff consultation all emerged in the analysis.

*“You know a couple of years ago with the previous manager, everything was unilaterally implemented. As in, it was put forward and we had to strictly abide by it irrespective of what we felt the outcome was going to be. There were several instances where you had to accept what was told to you and consequently, there was very little room for discussion or negotiation.”* [Healthcare provider]

When working in a small ED with limited staff turnover and a long-standing team who are familiar with daily routines and operations, it was deemed integral for managers to involve and engage frontline ED staff in the decision-making process while also managing strategies for running the department. Failure to give staff autonomy in their roles was anticipated to be a barrier to a successful implementation within this framework.

*“The emergency department was say anywhere from 98-99% senior. So, when you got a small department that is pretty much occupied by senior staff, it runs itself. Most of us have been nursing for 30 plus years. So, we know how the system works; we know what we have to do; we know how to solve problems; we are familiar with critical thinking to get issues resolved. However, this other manager was always critiquing us, and certainly not in a constructive manner”.* [Healthcare provider]

Amplifying these issues was the fact that there was a history of struggling with unapproachable, autocratic and unavailable managers in the ED. It left the clinicians with sentiments of neglect and varying overdue demands and expectations. This in-turn caused a “toxic environment” which was percived as a critical barrier to the successfull implementation:

*“But it really was like I said before, a toxic environment which placed everybody in on a defensive stance at all times and people did not want to go to work and more crucially, people did not like to work. If they did statistics on it, I am sure there was a huge spike in sick leave as people were just not wanting to go to work. That's the bottom line.”* [Healthcare provider]

### Available resources

Availability of resources was considered as a critical facet for the implementation of SurgeCon. As such, disparate resources, that crossed human and medical resources and several other silos (e.g., space constraint), were anticipated to be necessary considerations to ensure the long-term tenability of the SurgeCon intervention. Participants at all four EDs unanimously identified excess workload, and staff shortages, and absence of opportunities to ease workloads as the most significant anticipated barriers to implementation. To incorporate the new implementation system, not only was it asserted that all clinicians need to be available and have sufficient time to attend a staff training program, but they also need to regularly entering and updating SurgeCon data. Virtually all participants anticipated that the lack of human resources (i.e., insufficient medical staff) would be a crucial barrier.

*“Human resources can be a bit harder to come by because nurses are often treated as a commodity. There is so much overtime at the current time and requires increased staff.”* [Healthcare provider]

*“I think more family doctors are needed to lower the congestion in the ED.”* [Patient]

*“Need more staff. Patient asked multiple times to be taken to the bathroom after being left alone in a wheelchair... She asked again hours later and received no help so she peed in her wheelchair fully clothed and left without seeing a doctor due to embarrassment and such a lack of help.”* [Patient]

*“if we don't have enough staff or if we don't have enough beds. To me it don't matter what you're doing, it’s not going to work. It's going to be harder for it to work if you don't have the resources.”* [Healthcare provider]

Other than staff shortages, high staff turnover rates were cited as another anticipated barrier to implementation. The high level of staff turnover adversely impacted the level of communication among staff, and was also perceived as a significant challenge with regards to training and accommodating necessary implementation activities.

*“We have a lot of new nurses that are just coming out of program. So, helping mentor them with an overwhelmed emergency department is difficult as they are also trying to get their footing within the emergency department, and learn new skills and tasks. I find communications a bit lacking right now because we have so much new staff and they're just trying to get their footing and learn. In doing so, it is hard to have that communication. Like everyone helps wherever they can but you're also trying to, within that time, train your new staff as well. It's kind of a bit hectic.”* [Healthcare provider]

*“Rapid turnover of staff at HSC. So some of the staff have been through process improvement while many others have not.”* [Observer]

Insufficient admission space (e.g., inadequate number of beds) and the lack of physical space and rooms in EDs were often identified by clinicians as the primary cause of backlogs and overcrowding in EDs. These factors were anticipated to be barriers to the implementation process as they affect patient admissions, transfers, discharges, as well as the restructuring of the ED organization and workflow.

*“Some of the barriers would certainly be the inability to have free or vacant beds to transfer patients out of or transporting patients out of our department to a tertiary care facility.”* [Healthcare provider]

*“There needs to be more beds and seating arrangements.”* [Patient]

*“There is no current space adequate enough to run the flow center model.”* [Observer]

*“Rooms are sticky at times; space is small and overpopulated.”* [Observer]

### Communications and Networks Across the Organization

In order to ensure the successful adoption of SurgeCon, intra and inter-departmental communication was deemed to be a critical factor. Consistent and frequent communication between clinicians, particularly among physicians and nurses, is necessary to execute implementation activities successfully. However, this theme received mixed evaluations by participants. Poor communication and fragmented relationships between nurses and physicians, and lack of teamwork among staff emerged as significant barriers to the implementation of SurgeCon. In all four EDs, it was observed that physicians and nurses do not have any formal joint meetings and there was scarce communication between different units within EDs. The lack of shared multidisciplinary meetings in EDs decreased the chance of developing mutual understanding and commitment, building empathy and awareness toward each other’s challenges, and enhancing unity and teamwork.

*“There seems to be a huge miscommunication between staff, mainly to do with rules surrounding COVID.”* [Patient]

*“We do not sit down at the same table. There are family practice meetings, there are student emergency doc meetings and then, there are nursing meetings; you are not set at the same table. So, I cannot realistically know, feel nor empathize with anybody else’s needs if I am not even aware of them. We are never really made aware of that stuff.”* [Healthcare provider]

*“More communication between staff and patients would be very useful as most people will be more patient and understanding.”* [Patient]

Even in the case of personal conflicts and tensions arising between nurses and physicians, formal meetings of managers were considered as a predominate strategy to resolve the respective issues rather than directly involving staff. While the lack of intergroup (i.e., nurses and physicians) communication was evaluated as a barrier, participants positively evaluated intragroup communication, citing regular weekly formal meetings and informal daily meetings when necessary. Furthermore, nurses at one of the sites participated in a Facebook group to share their concerns.

*“There is a Facebook group… it was outlined that they are short a nurse, and they are looking for an extra nurse to come in. So, they posted that on the Facebook group in hopes that somebody will see it and come to their rescue.” [Healthcare provider]*

In general, a collaborative, supportive, receptive and cooperative environment were considered as a facilitator to implementation. The staff valued a culture of support, transparency, and availability. Also, it was assessed that working in a small ED, where the clinicians are familiar with one another more intimately and for a prolonged duration of time, positively fostered teamwork and supportive communication.

*“One main ED unit and there seemed to be good communication and in the smaller sites its quite easy to communicate”* [Observer]

Another barrier under this construct was identified as the lack of communication and dialogue between staff in two different units within the EDs. As these units operated independently, the minimal contact and communication between them became routine. Communication between the two units was restricted to the end of the shift and pertained primarily to handing-over patients. When problems arose, the most common means of communication to resolve or discuss the issue was conducted via email.

*“We’re taking care of the patients in unit one or unit two, and someone else is taking care of the patients in the other unit. So, I don't really talk to the other person. So, the only time when we communicate is around handover. So that's often sort of one we're saying, “Well, I am leaving, so you take over this patient.”* [Healthcare provider]

*“When we asked staff if they felt the areas of the departments communicated well together they said yes but while we watched it certainly seemed like all the areas functioned independently of each other. NO situational awareness.”* [Observer]

A common concern among participants pertained to the lack of engagement and involvement of other departments in the hospital in the implementation process of the intervention. The participants seemed to believe that the implementation could not be successful if other departments and stakeholders in the hospital have no intention to participate. Given the interconnectedness of a hospital’s departments, an intervention aimed to improve ED patient flow must also comprise meaningful engagement from external departments and must be prioritized at all levels of the organization rather than having the ED treated as an individual entity.

*“We've done a lot of improvements. For instance, our stroke process or STEMI process, those are things that we've implemented within our department to help streamline that category of patient, that were more focused on just the ED which were more successful. We haven't been able to be successful because of the barriers that lie outside of our department which are a little bit more systems or like, organizational wide. It becomes harder because maybe there's been an unwillingness to participate or not seeing the value because a lot of people don't see what it is like in our department all the time. So, they think that it's just value for us as opposed to value for them as well.”* [Healthcare provider]

Another potential barrier to implementation was the anticipated lack of physician participation in the implementation process. Nurses constantly emphasized the crucial role of physicians in the uptake of the intervention and furthermore desired assurance that the physicians will be well-informed about the implementation and will not be disengaged during the process.

*“I think physicians are older, more experienced positions or maybe just set in their ways and are less open to change. Some of the physician group will be more resistant.”* [Healthcare provider]

Despite the busy clinical environments, the success in the development and undertaking of the implementation hinged on constant and regular communication, including routine informal and formal meetings, that took place between the research team and clinicians. Although in-person meetings were preferable, due to COVID-19 pandemic related restrictions, videoconferencing was replaced to facilitate communication. Scheduling and arranging a meeting with clinicians because of the heavy workload, busy clinical schedule and demands was deemed as extremely challenging and proposed a critical barrier to implementation. Additionally, some of the research members do not have a direct line of communication with clinicians if not through internal facilitators or champions– i.e., nurse practitioners. Although a champion or facilitator demonstrated knowledge about the workload of clinicians which facilitated the scheduling of meetings, the lack of direct communication and in-person meetings seemed to be a critical barrier to implementation as the level of social engagement and connectedness between research staff and medical staff was adversely impacted.

### Previous intervention experiences

The previous experiences of staff members in implementing other interventions were evaluated as mostly positive by clinicians and researchers who conducted structured observations. However, some barriers were reported as well. The prior positive experiences of interventions were reported by the study participants, such as with X32 Healthcare’s Online Staffing Optimization project. In general, participants reported that the X32 project resulted in improved workflow efficiency, simplified and organized patient assessments, prioritized triage, and reduced wait times. These positive experiences with past interventions seemed to positively shape the participants perceptions of the SurgeCon implementation.

*“The X32 program was overall an effective program in my opinion. We did implement a lot of changes, overall infrastructure changes- the way that we introduce patients into our department and get them through the department to finally get them discharged. After the X32 program, we've seen dramatic improvements and changes versus the way that we were doing it.”* [Healthcare provider]

However, there were also negative perceptions of past intervenstions, for example, a lack of communication between researchers and staff, and the lack of follow-up evaluations to meet the contextually specific needs of the EDs.

*“Initially, there was a fair bit of communication between staff, the researchers and the end users but after it was implemented, I don't think there was any follow-up or any review of the X32.”* [Healthcare provider]

The perception of inadequacies or unsuccessful outcomes from prior intervention efforts appeared to influence the study participants' perceptions of the implementation of SurgeCon and was seen to be a potential barrier to future implementations. This historical context of past initiatives not meeting their intended goals created scepticism and resistance towards embracing the new SurgeCon program.

*“SurgeCon is new to us, but we've tried lots of different things over the years, and they've all failed. We've all put work into it… we'll try something, and we'll get all motivated to do it- we'll try it for six months, and everything that we've done falls apart inevitably.”* [Healthcare provider]

*“Many previous wait time related interventions over the past number of years and front line staff report mostly failures with staff reverting to old ways.”* [Observer]

### Need for change

Tension for change is considerd as an important concept for leaders seeking to improve performance in their organizations. It is a mechanism that created the energy and motivation needed to mobilize human beings into action. Although dissatisfaction with the current approach was the most common perspective as described from patients and providers in four EDs; this was considered concurrently as a strong motivation and potential barrier for clinicians to actively engage in the implementation process. Dissatisfaction with long wait-times and poor workflow was perceived as a major aspect of motivation; the most endorsed facilitator was found to be the perception of necessity of the intervention to rectify deficiencies in wait-time and workflow efficiency. Clinicians valued the change and deemed it as urgently necessary and beneficial. They valued the intervention and possessed an intrinsic inclination towards change as they had long-lasting concerns about the wait-time and workflow; they anticipated that SurgeCon might help to resolve the issues faced in EDs. Thus, clinicians in these EDs collectively valued the intervention and demonstrated an appreciation for the actions taken, which was seen to be one of the more crucial facilitators and implementation drivers.

*“I had to wait for 7 and a half hours which felt ridiculously long, even though there were not a lot of other people waiting.”* [Patient]

*“We have been waiting for 2 days because there were no in-patient beds available.”* [Patient]

*“The most important motivation is improving the quality of management for the patients and then, that will be reflected to the wellbeing of the patient as well as the smooth flow of the patient within the department. So, if there is any new idea that can facilitate this- they usually are very eager to adapt and undertake it.”* [Healthcare provider]

The participants frequently felt that the staff struggled to deal with the confusion arising from technological limitations in communicating information about wait times and the availability of medical resources. Several complaints were made regarding complications in scheduling appointments, inconsistent wait times, and misallocation of scarce resources which diminished the overall efficiency of the ED. These issue was considered motivating factors for the implementation of SurgeCon.

*“The sites lacked a digital patient tracking system that resulted in communication lapses between units.”* [Observer]

*“[Our province] is far behind in technology compared to other provinces.”* [Patient]

Participants expressed some dissatisfaction with the planned implementation as a result of not having enough time to participate, staff shortages, and heavy workloads. Two of the selected EDs were found to be particularly affected by this issue, which posed a significant obstacle even before the implementation which involved conducting pre-implementation in-depth interviews. The implementation of the quality improvement program would go ahead as planned, albeit with poor engagement and support from ED staff. Consequently, this lack of involvement might hinder the intervention from reaching their full potential.

*“I think that's going to be the biggest challenge is just getting them on board. Just the word “change” or “implementation” right now is a bit challenging.”* [Healthcare provider]

*“I mean morale in the past few years… it’s not in a good place and I think it's because of the increased business, and staff feel like they're burning out, so it's not that they don't do a good job. We need more resources.”* [Healthcare provider]

Two of the EDs chosen for this study had rejected previous intervention attempts, (e.g., X32 Healthcare’s Online Staffing Optimalization), which implies that the organizational climate might not be change-oriented. This phenomenon, other than dissatisfaction, was rooted in being resistant to changes (including technological changes) while conforming to the existing status-quo and being reluctant to adopt the consulted changes suggested from outside of the organization. To the participants, interventions meant novel systems, processes and skills which inherently implied altering the quondam workplace routine to adopt a newer system. While ED staff constantly struggled with the internal forces for change (e.g., heavy workload, staffing issues, and long wait time), they were not receptive to the external research team’s attempts at initiating change through the implementation of the intervention. This extended to not only external stimuli for change, but also propositions for change initiated by insiders which were not mobilized in either of the urban sites.

*Repeated resistance to technological changes expressed by staff in general.* [Observer]

*“It was unknown- you hear this company from outside is going to come in and fix your emergency department. A lot of people felt like, ‘Well, why do we need an outside company? Why don’t they just speak to the staff that actually works there to see how they could fix it?’ We knew what needed to be fixed but I kind of felt amused as to why did an external entity do it when they didn't ask the people that worked in a department first.”* [Healthcare provider]

*“I feel like change is a big thing for people personally and professionally. So, it is just going to take a while for people to get used to it and, it's something new that’s breaking our old routine of how we did things. I feel those will be some barriers. Technology is going to be a challenge and like I said, it’s a big change.”* [Healthcare provider]

During the pandemic, it became evident that engaging ED staff in implementation activities across all four EDs will create a challenging environment. Frontline staff had to manage exhaustion, frustration, burnout, isolation, and a higher volume of sick patients, making change initiation difficult. Clinicians often lacked the energy to participate in pre-implementation interviews, despite compensation and other offered incentives. In describing their experiences, one participant states:

*“We're just basically keeping our heads above water at this point.”* [Healthcare provider]

Low motivation to participate was caused due to feeling burdened by a heavy workload, COVID-19 regulations and subsequent procedure alterations. Thus, these dismayed clinicians struggled with the pandemic and thereby, served as another major barrier to the intervention.

*“With this pandemic, there's constant policy changes, procedure changes, and they're frustrated with it. So, if you want to bring in something else, even though it's going to help them a lot of times- they're resistant because it's just something else on their ‘To Do List’ and they don't want to be bothered with having to learn something else.”* [Healthcare provider].

## Discussion

### Summary of findings

Given the high rate of failure in translating evidence into practice in health care services and the challenges of implementing eHealth interventions [33, 34], it is necessary to assess barriers and facilitators prior to implementation to attain a successful implementation. This study found five facilitator-barrier pairs that were perceived to influence the successful implementation of SurgeCon in the four EDs in our study.

Management and leadership structures were the first facilitator-barrier pair. Such structures play a critical role in the integration and maintenance of innovative implementations in hospital settings[35]. The findings of Bonawitz et al. (2020) suggest that ineffective management and leadership serve as barriers to change in healthcare institutions [36]. Management systems that effectively encourage the involvement of health care providers in making ED-related decisions and support proactive managers are perceived to be crucial facilitators, as evidenced by the findings of this study, while disengaged managers and lack of staff autonomy are perceived as critical barriers. The findings observed in this study parallel those observed by Manca et al. (2018) [37], who found that participative leadership, which seeps into control-oriented management, poses a significant barrier to the dynamics presented by the organizational culture toward change. Furthermore, the lack of top-management sponsorship and presence-based culture presented a recurring barrier to the adoption of innovation in healthcare institutions. Our data suggest that early engagement of managers in implementation procedures and applying a participative leadership style that promotes active engagement of staff may facilitate successful implementation. This is supported by Bonawitz et al. (2020) who found a participative leadership style to be a critical component in successfully implementing change in a healthcare setting [36].

Available resources is the second facilitator-barrier pair. According to de Wit et al. (2018), implementing system-wide changes requires substantial prerequisite committed hospital resources [38]. However, tailoring a strategy may permit circumventing change management projects that require committing substantial additional resources [39]. Furthermore, Barnett et al. (2011) express that the influence of human-based resources is integral in the process of developing, establishing, and diffusing innovations in healthcare institutions [40]. However, the Canadian Institute for Health Information (2021) points out the stark shortages and increasing staff turnover rates in medical staff within the Canadian healthcare system [24]. With a perpetually changing and constrained workforce, any pursuit to adopt an implementation will intrinsically face initial challenges. Additionally, de Wit et al. (2018) provide a comprehensive overview of the critical resources prior to initiating change: depending on the idiosyncratic details of implementation, educational resources need to be made available (with minimal barriers to accessing them), along with committed hospital resources in the form of financial, staffing, and other resources [38]. Furthermore, a lack of medical resources negatively impacts patient admissions, patient transfer delays, cancellation of surgeries, or early discharges [41]. Inadequate financial, technological, human, and medical resources were consistently identified as anticipated barriers across all four ED sites. Although implementing SurgeCon does not require substantial additional resources and the ED sites are provided with the technological equipment and educational requirements prior to the intervention, the shortage of medical staff and lack of medical resources remain potentially significant barriers, as found by this study.

The third facilitator-barrier pair is communications and networks across the organization. Considering the insights gained from previous studies on leadership structures in healthcare institutions, communication is a critical symptom of a participative leadership structure [35, 36, 42, 43]. It is repeatedly established that teamwork, trust and other parameters of the respective organizational climate are founded by the principles of the underlying leadership structure. According to our study however, even in the participative leadership structure which embraces engagement and involvement of staff, ED environments suffer a lack of communication between nurses and physicians and between different ED units. While the minimal formal and informal discussions that occur between physicians and nurses may meet the basic requirements for professional standards, they are not fully cognisant of each other’s concerns and challenges. To fully engage and participate in the implementation of an intervention, collaboration between all ED staff is required. Lack of communication, dialogue, and teamwork among staff is recognized as an anticipated barrier to successful implementation. Conversely, constant communication and dialogue between the research staff and healthcare provider is considered as a practice that would facilitate the intervention’s implementation. However, in our case, due to COVID-19 restrictions, almost all communications were transferred from in-person to a virtual medium. Being overwhelmed by COVID-19 regulatory demands, staff shortages and burdensome workloads, clinicians were not left with enough energy and time to participate in pre-implementation on-line interviews.

The fourth facilitator-barrier pair, previous intervention experiences, were also anticipated to impact the SurgeCon implementation. Hamilton et al. (2010) found that prior experience with change efforts contributed to readiness for change in healthcare institutions [44]. As such, it is expressed that previous experience with interventions contributes to calibrating an appropriate organizational climate that is conducive to change. Previous experience greatly assists in establishing the appropriate steps and instilling confidence to create a ripe organizational climate for the implementation [45]. Zapka et al. (2013) express the need for reviews of past experiences of change as a necessary element to sustain the implementation [46]. The findings of this study with regard to previous experience of interventions and its potential to make a positive or negative impact on future interventions parallel those observed by previous scholars. Our data reveals that the negative perceptions of past intervenstions (e.g., lack of follow-up evaluations), was considered a notable obstacle to the implementation of SurgeCon.

The fifth facilitator-barrier pair was the need for change. Grol (2013) illustrates the importance of the perception of necessity in successfully adopting an intervention, particularly in a healthcare environment. Institutions with a positive perception of the necessity of an intervention are more likely to adopt and sustain an implementation [47]. Tension for change in implementation science is defined as the proclivity for shareholders to perceive the current situation as requiring a change or intolerable [48–50]. Our findings illustrated that dissatisfaction with the current system, with long wait times and poor workflow in EDs, was perceived as a necessity for urgent change and intervention. However, the perception of the necessity of the intervention does not necessarily imply valuing or practicing the change requirements. Our study supports findings of the inverse relationship between staff burnout and motivation to support an intervention [51, 52]. When considering the drastic national rise in burnout experienced by healthcare workers in Canada [53], the current healthcare environment is not conducive to change. Lack of time, staff shortages, and heavy workload coupled with COVID-19 fatigue and burnout did not leave clinicians with sufficient energy to even participate in pre-implementation interviews, let alone in interest in being actively involved in the intervention. Additionally, this study found that using new technology and altering the workplace routines were perceived as barriers to change among clinicians. Regardless of the high level of dissatisfaction and staff workload, clinicians were still resistant to the interventions proposed by external sources.

### Strategies for overcoming barriers and enhancing facilitators

Identifying and evaluating barriers and facilitators alone is only the first step in enhancing the probability of successful implementations of eHealth interventions such as SurgeCon. It is also important to formulate a set of strategies for hospitals to overcome the identified barriers and enhance the facilitators (Fig. 1). The recommended strategies—staff training, frontline champions, performance data review, communicating the value of the intervention, encouraging active engagement of ED staff, assigning an individual to regularly record data, and requiring capacity analysis—aim to address and overcome barriers while capitalizing on facilitators. These multi-faceted strategies were identified through discussions with decision makers, clinicians, patients, and research team members as well as lessons learned from SurgeCon’s implementation at the pilot site.

To elaborate on the specific components: It is crucial that a majority of ED staff attend a training on paitient flow and have ED leadership participate in software configuration to adjust and tailor SurgeCon’s the digital eHealth platform to their ED. Attending training sessions facilitates the adoption of quality improvement initiatives and patient flow strategies included within the SurgeCon platform and encourages ED staff to become actively engaged with the implementation process. This process is essential to foster an active participation and discussion between all tiers of staff which may not routinely transpire. The training course needs to actively engage frontline staff and must include the following modules: Interactive Simulation, SurgeCon eHealth Platform, and Patient Centeredness modules. The aim of the Interactive Simulation module is to provide insights into the rationale of connecting the software to process improvement and elucidate its procedure in a practical setting using ED-based scenarios. Since the module will be interactive, it allows for greater clarity to ensure that learning outcomes are achieved. The SurgeCon eHealth Platform module will assist ED staff in becoming familiar with the digital whiteboard application. This includes learning how the system collects and reports information, how to interpret and respond to system notices and warnings, and how to customize the dashboard to create a site-specific, adaptive version of SurgeCon that addresses the unique needs of their ED. The Patient Centeredness module comprises an educational session which reinforces the core importance of values pertaining to patient care across the following topics: providing quality ED care to all patients regardless of urgency; treating patients with respect; and considering the patient’s visit to an ED as always of vital necessity.

Having a dedicated frontline champion who is selected by ED management and trained by the implementation team can help ensure effective communication and facilitate the implementation process. These individuals can act as a liaison between ED staff and the research team, providing ongoing support and addressing any questions or concerns that may arise. In addition, they can provide valuable feedback to the research team regarding technical issues or challenges encountered during implementation which can help inform adjustments and improvements to the intervention. Ultimately, having frontline champions who are invested in the success of the intervention can contribute to a more seamless and effective implementation process.

Continuous performance reporting plays a crucial role in enhancing the operational efficiency and effectiveness of EDs and contributes to the development of improved operational strategies by providing meaningful data. In this study, the research protocol involves prominently displaying department-level data in the ED, such as at nursing stations, and providing individual-level performance reports to physicians at the participating sites. However, in the post-COVID era, EDs have been experiencing staffing shortages, which have necessitated changes in the reporting protocols of this study, particularly regarding key performance indicator (KPI) data. The KPIs examined in this study include the time to physician initial assessment (PIA), the length of stay in the ED (LOS), and the rate of patients leaving the ED without being seen by a physician (LWBS). These KPIs are widely recognized as the gold standard for evaluating ED performance. However, these indicators assume consistent operating conditions, and the reliability of using them as the primary method for assessing department efficiency diminishes in the presence of staff shortages. Providing individual physicians with performance reports may serve as a reminder of the operational challenges they have faced rather than providing a fair assessment of their ability to efficiently manage patient flow in their department. As a result, the research team decided to recommend aggregated department-level performance reports. Ultimately, the primary goal is to increase physician motivation to utilize SurgeCon by demonstrating its capacity to reduce door-to-doctor time, which is a critical metric for assessing standards of emergency care and efficiency.

It is important to the research team to communicate the importance and value of SurgeCon by presenting a successful implementation in the pilot site to raise awareness about the prospective results and enhance motivation for the adoption of the intervention. Additionally, implementing interventions is a “collective action” which necessitates a commitment to the process by all members. As Weiner (2009, p. 2) [54] states “implementing complex organizational changes involves collective action by many people, each of whom contributes something to the implementation effort […] problems arise when some feel committed to implementation, but others do not.” To stimulate engagement, compensation (i.e., full payment for attendance including travel and meals) offers for participating in training sessions and interviews; refreshments, in the form of snacks and beverages, were also provided at every training session. Furthermore, assigning an individual whose primary role is to manually enter data that cannot be automated into SurgeCon’s eHealth system, and using demand and capacity analysis to determine staffing models that will benefit the department are among the suggested strategies to overcome several of the encountered barriers to implementation.

## Conclusion and implications

Successfully implementing eHealth systems goes beyond addressing technological aspects alone. It requires a thorough exploration of potential barriers and facilitators and the development of strategies to overcome barriers and enhance the facilitators. SurgeCon aims to enhance quality standards, improve efficiency, and increase satisfaction among both patients and providers in EDs. However, implementing such a quality improvement initiative in EDs presents challenges. Therefore, identifying these barriers and facilitators is crucial for developing tailored implementation strategies that are contextually relevant. This approach helps to ensure a smooth and sustainable transition, leading to long-term success and optimal performance. This study extends the findings in relevant literature by indentifying these facilitator-barrier pairs and providing a set of strategies to overcome the barriers and enhance the facilitators in the implementation of a large-scale quality improvement program. In investigating the factors associated with the successful adoption of SurgeCon, a broader consideration of the barriers and facilitators can be derived. Understanding these factors can assist in identifying obstacles and motivators that enable the sustainability and effectiveness of interventions at other EDs; this is critical given the high failure rate of ED quality improvement programs.

Effective management and leadership structures and participative leadership styles that encourage staff involvement and proactive management may facilitate ED implementations. Emphasis on the allocation of sufficient hospital resources (i.e., technological, human, and medical) and effective communication and collaboration are essential for fostering a supportive and cohesive work environment, thus facilitating such interventions. Those with positive perceptions of the need for the intervention are more likely to adopt and sustain implementation efforts, and previous experiences with interventions and the perception of the need for an intervention emerged as influential factors in the readiness for change.

This study strategically incorporates triangulation. By doing so, it addresses inherent blind spots and biases in each method, enhances the validation of data, and offers diverse perspectives on the topic. This triangulation not only validates findings but also contributes to a more comprehensive and calibrated understanding of the phenomena under investigation. Furthermore, this study involves a multi-disciplinary planning and implementation team to comprehensively study the various facilitators and barriers prior to implementation.

This study, like any rigorous research endeavor, is not exempt from limitations, and it is essential to openly acknowledge these factors to provide a transparent understanding of the study's scope. While our study gains insights from four diverse EDs, it is crucial to note a limitation in its context-specific nature. Our primary focus revolves around understanding barriers and facilitators before implementing the SurgeCon quality improvement program in Canadian EDs. Findings may lack broad generalizability. However, our emphasis on transferability urges researchers to assess the applicability of insights in similar settings, fostering a nuanced understanding. In this study, the data collector observed potential social desirability tendencies among participants. To address this, we made efforts to assure participants of anonymity and confidentiality, provided clear communication about the study's purpose and data use, and incorporated strategies like follow-up questions. Additionally, we encouraged participants to share examples to illustrate their responses, aiming to mitigate potential response bias [55]. Finally, the study, conducted within a specific timeframe, must consider the dynamic healthcare landscape. The advent of COVID-19 brought rapid changes to healthcare policies, ED protocols, and overall healthcare delivery. Acknowledging this evolving context during and after data collection is crucial for interpreting the study's findings in the broader context of a changing healthcare system.

The findings of this study will guide future initiatives for the implementation of quality improvement programs within the complex environment of EDs by identifying facilitators and barriers prior to implementation to ensure they are continually considered during the design phase of an intervention. We propose that it is important to examine these factors before implementing such systems so that the implementation can be designed and managed to address the multivariate impact they may impose.

### Supplementary Information

Supplementary Material 1.

## Data Availability

The datasets generated and/or analysed during the current study are not publicly available to protect the confidentiality of participants’ data but are available from the corresponding author upon reasonable request.

## Abbreviations

ED: Emergency department
CFIR: Consolidated Framework for Implementation Research
NL: Newfoundland and Labrador
OR4KT: Organization Readiness for Knowledge Translation
KPI: Key performance indicator
PIA: Physician initial assessment
LOS: Length of stay
LWBS: Leaving the ED without being seen
